# Finding the right sequence of drugs

**DOI:** 10.7554/eLife.72562

**Published:** 2021-09-09

**Authors:** Anh Huynh, Kevin B Wood

**Affiliations:** 1 Department of Biophysics, University of Michigan Ann Arbor United States; 2 Department of Physics, University of Michigan Ann Arbor United States

**Keywords:** *Pseudomonas aeruginosa*, antibiotic resistance, sequential therapy, evolutionary medicine, collateral sensitivity, cellular hysteresis, Other

## Abstract

Rapidly switching between similar antibiotics may help to slow down the evolution of resistance.

**Related research article** Batra A, Roemhild R, Rousseau E, Franzenburg S, Niemann S, Schulenburg H. 2021. High potency of sequential therapy with only β-lactam antibiotics. *eLife* **10**:e68876. doi: 10.7554/eLife.68876

In order to survive, many living organisms need to be able to adapt to their ever-changing environment. These past experiences shape the behavior of creatures big and small – from mutations in single genes to neurological changes that underlie memory formation in primates.

There is substantial evidence that environmental history affects how bacteria respond to antibiotics (Barbosa et al., 2019; Card et al., 2019; Nichol et al., 2019; Santos-Lopez et al., 2019; Yen and Papin, 2017). This has led researchers to suggest that switching between drugs over time could help slow down antibiotic resistance, as this will force the bacteria into a scenario where their existing solutions (resistance to the current drug) cannot protect them from tomorrow’s problem (a new drug). However, this method has led to mixed results (Abel zur Wiesch et al., 2014; Imamovic et al., 2018), and optimizing this approach is challenging, in part because it is unclear which features of the antibiotic sequence are the most important and guarantee the best results.

Now, in eLife, Hinrich Schulenburg (University of Kiel and Max Planck Institute for Evolutionary Biology) and colleagues – including Aditi Batra and Roderich Roemhild as joint first authors – report the results of experiments on the multi-drug resistant bacteria *Pseudomonas aeruginosa* (Batra et al., 2021). The team (who are based in Austria and Germany) exposed the bacteria to various sequences of three antibiotics that belong to commonly used classes of drugs: one class targets the ribosome, one targets DNA gyrase, and one targets the cell wall. In some cases, three drugs from different classes were used (that is, a heterogeneous sequence), and in some cases all three drugs belonged to the same class (a homogenous sequence). Batra et al. also varied the temporal properties of each sequence by switching between the drugs rapidly, slowly, or in a random order. The growth rate, phenotypic resistance levels and population genetics of the evolved populations were then analyzed to determine which sequences of drugs were the most effective at eliminating the bacteria (Figure 1A).

**Figure 1.**
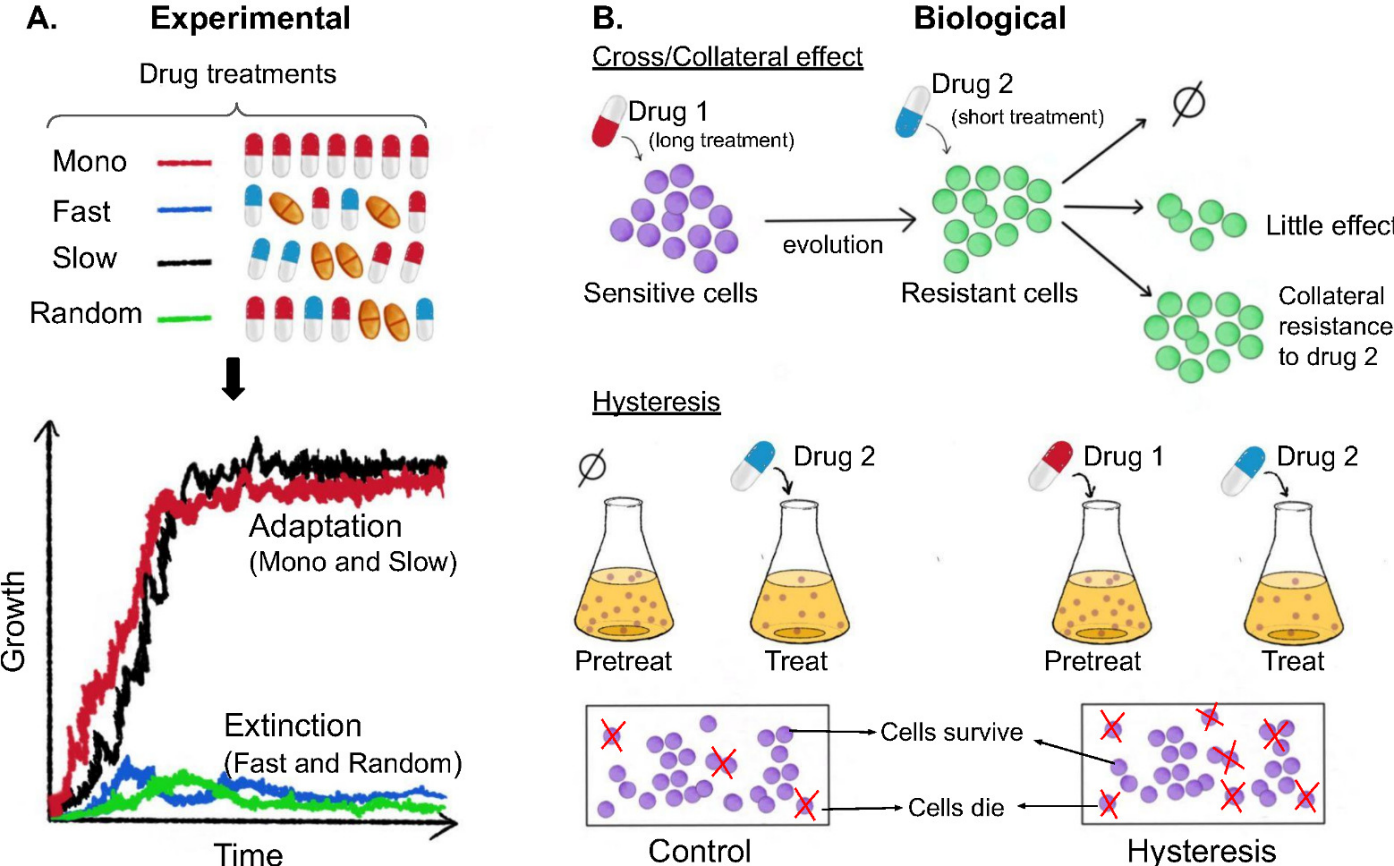
Experimental and biological features of effective drug sequences. (**A**) Batra et al. applied different sequences of antibiotics to 756 populations of *P. aeruginosa *(top panel). The bacteria were treated with either a single drug (monotherapy; row 1), or three antibiotics which were switched rapidly (row 2), slowly (row 3) or in a random order (row 4): the three drugs were either from the same class (homogeneous) or from different classes (heterogeneous). This experiment revealed that fast (blue line) and random (green line) switching between three homogeneous beta-lactam drugs reduced bacteria growth and resulted in higher levels of extinction (bottom graph). (**B**) The effects of the different sequences are also impacted by biological features. (Top panel) When sensitive bacteria (shown in purple) are treated with the first drug, some cells will evolve genetic changes that make them resistant to the antibiotic treatment (shown in green). These evolutionary changes can lead to collateral effects that make the bacteria less (top arrow), equally (middle arrow) or more (bottom arrow) resistant to the second drug. (Bottom panel) Treatment with the first drug may also lead to negative hysteresis, when short-term physiological changes enhance the bacteria’s response to the second drug (right), leading to more cell death in the population compared to bacteria not pre-treated with the first drug (left).

Image credit: Anh Huynh.

In addition to these experimental parameters, the impact of different antibiotic sequences could also depend on how the population biologically responds to consecutive drug exposures (Figure 1B). For example, the genetic changes bacteria evolve in response to one antibiotic can lead to collateral effects that increase the population’s resistance or sensitivity to another drug. Because collateral resistance occurs more frequently between drugs of the same class, heterogeneous sequences of antibiotics are thought to be more effective at eliminating bacteria (Imamovic and Sommer, 2013; Lázár et al., 2013; Maltas and Wood, 2019; Pál et al., 2015; Lázár et al., 2014). Treatment with unrelated drugs has also been shown to favor negative hysteresis, which is when short-term physiological changes induced by one antibiotic enhance susceptibility towards another. Indeed, a recent study found that rapid switching between antibiotics from different classes promoted extinction of bacterial populations, even when the drugs were used at sub-inhibitory levels (Roemhild et al., 2018).

However, Batra et al. found that homogenous sequences of beta-lactams (a class of antibiotics that target the cell wall) were surprisingly more effective at clearing bacteria. The experiments also revealed that extinction tended to occur early in the treatment and was less effective when drugs were switched more slowly. A particular heterogeneous set of drugs also tended to not eliminate bacteria, indicating that heterogeneity, alone, does not guarantee success.

So, what are the important characteristics of a ‘good’ antibiotic sequence? To answer this question, Batra et al. used a common class of statistical models to probe for signatures of successful sequences. They found that extinction was strongly favored by two biological properties — low rates of spontaneous resistance and low levels of collateral resistance — and was also enhanced when switching between drugs was fast or random. This suggests that although thefindings of Batra et al. contradict the proposed benefits of using unrelated drugs, they still validate a portion of the underlying logic: using antibiotics with strong collateral effects and hysteresis enhances the impact of sequence therapy. It just turns out, however, that drugs with these characteristics are not always from different classes.

This study offers insight into how past antibiotic exposure shapes the response of bacterial populations to new challenges. In doing so, it provides a roadmap for future studies investigating how even the simplest organisms harbor signatures of past challenges and potential evolutionary solutions.

## References

[bib1] Abel zur Wiesch P, Kouyos R, Abel S, Viechtbauer W, Bonhoeffer S (2014). Cycling empirical antibiotic therapy in hospitals: meta-analysis and models. PLOS Pathogens.

[bib2] Barbosa C, Römhild R, Rosenstiel P, Schulenburg H (2019). Evolutionary stability of collateral sensitivity to antibiotics in the model pathogen *Pseudomonas aeruginosa*. eLife.

[bib3] Batra A, Roemhild R, Rousseau E, Franzenburg S, Niemann S, Schulenburg H (2021). High potency of sequential therapy with only β-lactam antibiotics. eLife.

[bib4] Card KJ, LaBar T, Gomez JB, Lenski RE (2019). Historical contingency in the evolution of antibiotic resistance after decades of relaxed selection. PLOS Biology.

[bib5] Imamovic L, Ellabaan MMH, Dantas Machado AM, Citterio L, Wulff T, Molin S, Krogh Johansen H, Sommer MOA (2018). Drug-driven phenotypic convergence supports rational treatment strategies of chronic infections. Cell.

[bib6] Imamovic L, Sommer MO (2013). Use of collateral sensitivity networks to design drug cycling protocols that avoid resistance development. Science Translational Medicine.

[bib7] Lázár V, Pal Singh G, Spohn R, Nagy I, Horváth B, Hrtyan M, Busa-Fekete R, Bogos B, Méhi O, Csörgő B, Pósfai G, Fekete G, Szappanos B, Kégl B, Papp B, Pál C (2013). Bacterial evolution of antibiotic hypersensitivity. Molecular Systems Biology.

[bib8] Lázár V, Nagy I, Spohn R, Csörgő B, Györkei Á, Nyerges Á, Horváth B, Vörös A, Busa-Fekete R, Hrtyan M, Bogos B, Méhi O, Fekete G, Szappanos B, Kégl B, Papp B, Pál C (2014). Genome-wide analysis captures the determinants of the antibiotic cross-resistance interaction network. Nature Communications.

[bib9] Maltas J, Wood KB (2019). Pervasive and diverse collateral sensitivity profiles inform optimal strategies to limit antibiotic resistance. PLOS Biology.

[bib10] Nichol D, Rutter J, Bryant C, Hujer AM, Lek S, Adams MD, Jeavons P, Anderson ARA, Bonomo RA, Scott JG (2019). Antibiotic collateral sensitivity is contingent on the repeatability of evolution. Nature Communications.

[bib11] Pál C, Papp B, Lázár V (2015). Collateral sensitivity of antibiotic-resistant microbes. Trends in Microbiology.

[bib12] Roemhild R, Gokhale CS, Dirksen P, Blake C, Rosenstiel P, Traulsen A, Andersson DI, Schulenburg H (2018). Cellular hysteresis as a principle to maximize the efficacy of antibiotic therapy. PNAS.

[bib13] Santos-Lopez A, Marshall CW, Scribner MR, Snyder DJ, Cooper VS (2019). Evolutionary pathways to antibiotic resistance are dependent upon environmental structure and bacterial lifestyle. eLife.

[bib14] Yen P, Papin JA (2017). History of antibiotic adaptation influences microbial evolutionary dynamics during subsequent treatment. PLOS Biology.

